# Joining up the scattered anticancer knowledge on auraptene and umbelliprenin: a meta-analysis

**DOI:** 10.1038/s41598-024-62747-z

**Published:** 2024-05-23

**Authors:** Mohammadhosein Shakiba, Fatemeh B. Rassouli

**Affiliations:** 1 Department of Biology, Faculty of Science, Ferdowsi University of Mashhad, Mashhad, Iran; 2 Novel Diagnostics and Therapeutics Research Group, Institute of Biotechnology, Ferdowsi University of Mashhad, P.O. Box: 9177948974, Mashhad, Iran

**Keywords:** Auraptene, Umbelliprenin, Anticancer, Meta-analysis, In vitro toxicity, Natural coumarin, Pharmacology, Cancer therapy, Machine learning, Statistical methods

## Abstract

Auraptene (AUR) and umbelliprenin (UMB) are naturally occurring prenylated coumarins that have demonstrated promising anticancer effects across various human cancer cell lines. This meta-analysis aimed to systematically assess, compare, and quantify the anticancer efficacy of AUR and UMB by synthesizing evidence from in vitro studies. A comprehensive literature search identified 27 eligible studies investigating AUR or UMB against cancer cells. Mixed-effects models revealed significant negative associations between coumarin dose and viability for AUR (est. = − 2.27) and UMB (est. = − 3.990), underscoring their dose-dependent cytotoxicity. Meta-regression indicated slightly higher potency for UMB over AUR, potentially due to increased lipophilicity imparted by additional isoprenyl units. Machine learning approaches identified coumarin dose and cancer type as the most influential determinants of toxicity, while treatment duration and the specific coumarin displayed weaker effects. Moderate (AUR) to substantial (UMB) between-study heterogeneity was detected, although the findings proved robust. In summary, this meta-analysis establishes AUR and UMB as promising natural anticancer candidates with clear dose-toxicity relationships across diverse malignancies. The structural insights and quantifications of anticancer efficacy can inform forthcoming efforts assessing therapeutic potential in pre-clinical models and human trials.

## Introduction

Cancer remains a critical global health issue, with escalating prevalence and mortality rates in both developed and developing nations. The most recent cancer data from GLOBOCAN 2020 reported approximately 19.3 million cases and 10 million fatalities. Notably, lung carcinoma stands as the most frequently diagnosed malignancy and the primary cause of cancer-related death, followed by breast, prostate, and gastrointestinal cancers^1^.

Recent advancements in technology have notably improved cancer diagnostic aspects in clinical management. However, when it comes to cancer treatment, unsatisfactory clinical outcomes persist due to the limited bioavailability and weak targeting of conventional treatments, alongside the innate or acquired resistance of cancerous cells^2^. These challenges underscore the pressing need for more efficacious therapeutic alternatives in clinical practice.

Natural products have gained considerable attention due to their significant anticancer activity. Plant secondary metabolites are promising sources for anticancer drug development, with favorable absorption, distribution, and metabolism. Moreover, the growing interest in exploring medicinal plants is attributed to their environmental sustainability and minimal side effects. Currently, over 60% of anticancer agents with clinical utility are derived from herbal sources, such as vincristine, vinblastine, irinotecan, topotecan, etoposide, podophyllotoxin, and paclitaxel^3,4^. Among the biologically active plant products, coumarins are particularly interesting because of their wide distribution in the plant kingdom and diverse biological activities^5^. Coumarins are secondary metabolites found in the leaves, roots, and seeds of plants belonging to the Apiaceae and Rutaceae families. They are composed of benzene rings, alpha-pyrone, and a carbonyl group connected to the C-2 of the pyrone ring. The conjugated double bonds of coumarins create a crucial electrical environment attributed to their free radical scavenging ability. In addition, the ability of coumarins to exert non-covalent interactions, like hydrogen bonds, metal coordination, and van der Waals force with many active sites of proteins, leads to their anticoagulant, anti-inflammatory, antibacterial, antiviral, antifungal, antioxidant, antihypertensive and antidiabetic effects^6^. The pharmaceutical effects of coumarins depend on various substitutions within their benzo-α-pyrone structure. For instance, prenylation, involving the addition of an unsaturated chain like prenyl, geranyl, or farnesyl side chain, enhances the lipophilicity of coumarins, thereby boosting their biological activity^7,8^.

Plant-derived natural coumarins exhibit potent anticancer properties through diverse mechanisms. These include inhibiting angiogenesis and microtubule polymerization, inducing caspase-mediated apoptosis and cell cycle arrest, regulating enzyme activity (e.g., carbonic anhydrase, histone deacetylase, telomerase, aromatase, topoisomerase, EGFR, and HER2 tyrosine kinases), acting as intercalating and alkylating agents and hormone antagonists, inhibiting signaling pathways like PI3K/Akt/mTOR and ERK1/2, and preventing/reversing multidrug resistance^9^.

**Figure 1:**
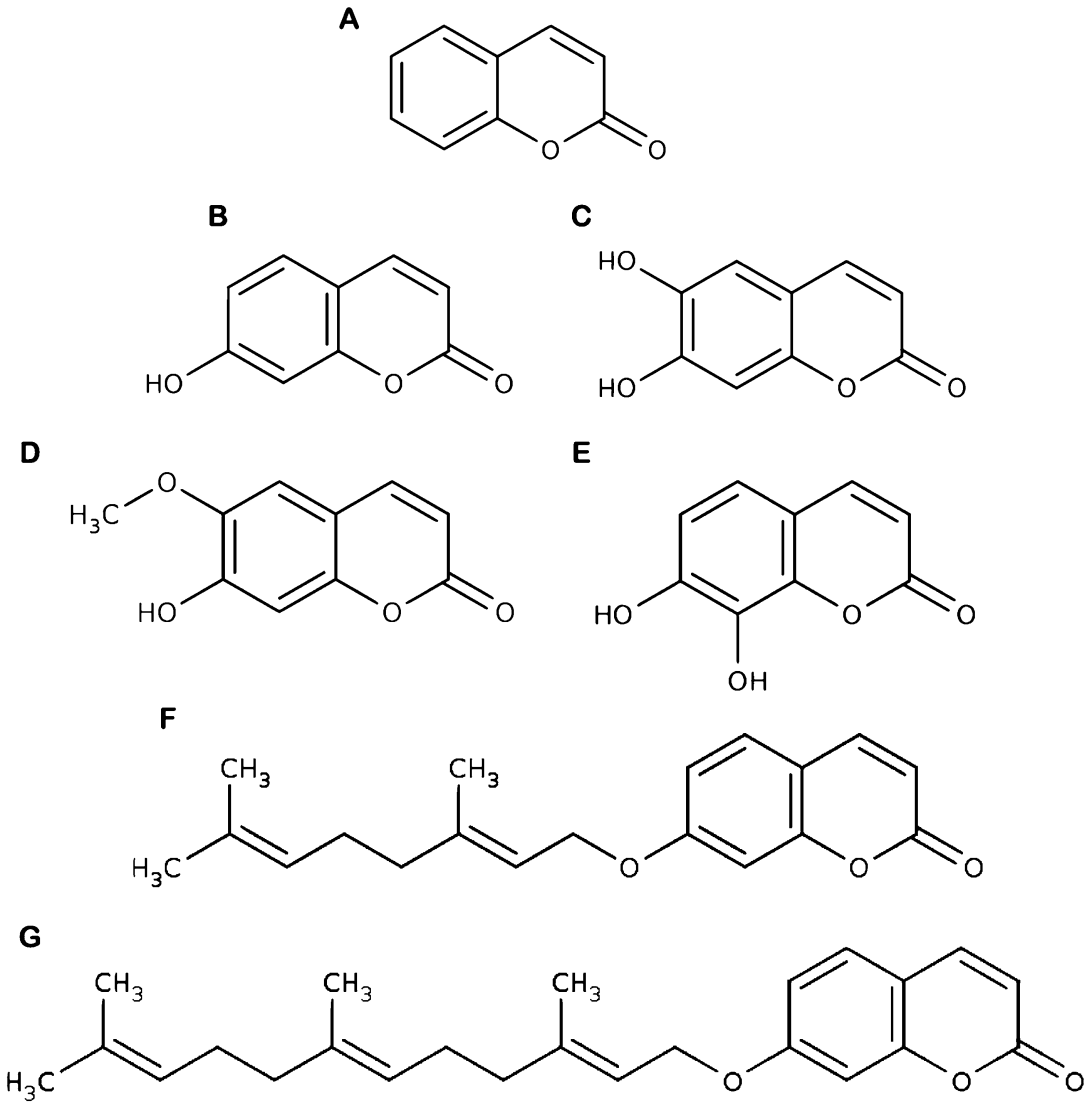
Chemical structures of coumarin (**A**), umbelliferone (**B**), esculetin (**C**), scopoletin (**D**), daphnetin (**E**), auraptene (**F**), and umbelliprenin (**G**). All structures are from PubChem.

Coumarins are categorized into four primary subtypes: simple coumarins, furanocoumarins, pyranocoumarins, and pyrone-substituted coumarins. Simple coumarins, such as umbelliferone (CID: 5,281,426), esculetin (CID: 5,281,416), scopoletin (CID: 5,280,460), daphnetin (CID: 5,280,569), auraptene (CID: 1,550,607), and umbelliprenin (CID: 1,781,413), are characterized by hydroxyl, alkoxyl, and alkyl substitutions on their basic structure, along with their glucosides. (Fig. 1).

Research on simple coumarins has demonstrated their potential anticancer properties. For example, umbelliferone has been found to exert toxic effects on various carcinoma cells, including those of the lung, liver, colon, prostate, and breast, primarily through the induction of cell cycle arrest and caspase-mediated apoptosis^10,11^. Similarly, esculetin has shown anticancer effects on hepatocellular carcinoma cells by blocking the cell cycle, inducing apoptosis, and increasing caspase activity^12^. Daphnetin, on the other hand, has been found to inhibit the proliferation of lung adenocarcinoma cells by inducing apoptosis and suppressing the Akt/NF-κB signaling pathway^13^. Lastly, scopoletin has been observed to inhibit the growth of prostate cancer cells by inducing cell cycle arrest^14^. These findings highlight the potential of simple coumarins in cancer treatment.

Auraptene (AUR) (C_19_H_22_O_3_) and umbelliprenin (UMB) (C_24_H_30_O_3_) are types of prenylated coumarins. In these compounds, the phenolic hydrogen is replaced by geranyl and trans-farnesyl groups, respectively. They are found in various fruits and vegetables, particularly those from the *Ferula*, *Citrus*, and *Punica* genera. The extraction and analysis of these oxyprenylated phenylpropanoids are typically performed using subcritical butane extraction in conjunction with ultra-high-performance liquid chromatography and solid-phase extraction methods. These techniques are not only safe and environmentally friendly, but they also provide high yields in a short time and maintain the chemical stability of the oxy-prenylated metabolites^15,16^.

AUR is the most prevalent geranyloxycoumarin found in nature. It is commonly sourced from fruits of the *Citrus* genus, such as grapefruit, lemon, and orange, as well as from *Ferula* species like *F. szowitsiana* and *F. assa-fetida L.*, *Eremurus persicus*, and pomegranate (*Punica granatum L.*)^15–19^. AUR is not only prevalent but also boasts a wide array of pharmaceutical properties. These include antioxidative, anti-genotoxic, antidiabetic, antibacterial, immunomodulatory, and cancer chemopreventive effects^20–22^. In recent years, the anticancer effects of AUR have garnered significant attention. This coumarin has been observed to reduce cell survival, proliferation, and migration while inducing apoptosis and improving chemosensitivity in several human cancer cell lines including glioblastoma, breast, colon, gastric, hepatic, prostate, esophageal, and ovarian carcinomas^19,23–34^. It is worth noting that the anticancer effects of AUR on leukemia, colon, gastric, and esophageal carcinoma cells have been compared with those of established anticancer drugs such as arsenic trioxide, doxorubicin, vincristine, cisplatin, paclitaxel, and 5-fluorouracil^24,25,27,35^. Moreover, studies in rodent models have demonstrated reduced tumor size and induced apoptosis in hepatic, gastric, colon, and skin cancers post-administration of AUR^26,36–38^. Mechanisms introduced for AUR anticancer effects include induction of caspases, P21, P53, CXCL3, BAX, down-regulation of cyclin D1, BCL-2, CD44, IGF-1, VEGFR, HSP27, ALDH, and GATA6, inhibition of COX-2 and iNOS, reduction of ERK1/2 along with G_0_/G_1_ cell cycle arrest and inhibition of PI3K-AKT-mTORC pathway^19,23,24,30,39^.

UMB, a naturally abundant geranyloxycoumarin, is found in various fruits and vegetables. These include *Citrus limon*, *Apium graveolens L., Angelica archangelica L., Peucedanum palustre, Amaranthus retroflexus L., Spinacia oleracea L., Lycium barbarum L., Chenopodium quinoa, F. persica* and *F. sinkiangensis*^40–43^. It has been studied for its remarkable chemical stability under various conditions, such as thermal, photochemical, oxidative, and hydrolytic stress, both in solution and solid form^42^. UMB exerts anti-inflammatory, antibacterial, antileishmanial, analgesic, and antioxidative effects through inhibition of matrix metalloproteinases and pro-inflammatory enzymes like 5-lipoxygenase, abolition of P-glycoprotein-mediated drug efflux and up-regulation of Mcl-1^21,44,45^.

Studies have additionally shown that UMB exerts anticancer effects on various types of human cancer cells, including those found in the skin, breast, lung, colon, and gastric tissues. These effects are achieved through multiple mechanisms, such as inhibiting the cell cycle at the G0/G1 phase and influencing key processes like migration, invasion, and angiogenesis. UMB accomplishes this by targeting important signaling pathways like Wnt, NF-ĸB, TGFβ, and Fox3.

UMB also regulates both the extrinsic and intrinsic apoptotic pathways via modulating various apoptosis-related proteins and genes, including P21, P16, BAX, pRB, caspases, Survivin, c-MYC, cyclins D and E, and CDK4 and CDK2^46–50^. Additionally, in animal models of breast, lung, colon, and gastric cancers, the administration of UMB inhibited tumor growth and induced anti-metastatic effects^41,51–53^.

Regarding the biosafety of AUR and UMB, several studies proved that both agents induced less toxicity in normal/healthy cells when compared with cancer cells^25,30,39,46,49,54,55^. Anticancer studies carried out in vivo reported that AUR and UMB showed no toxic histopathological effects on organ tissues and were considered safe agents for the immune system with no apparent drug-induced adverse effects^31,39,41,52,53,56–59^. Furthermore, studies on animal models of neurological disorders and cholestasis have revealed neuroprotective and hepatoprotective effects of AUR and UMB, indicating their safety and being well-tolerated in these animal models^55,60–62^. Noteworthy, the only randomized study in healthy volunteers using AUR enriched-extracts of peels of *Citrus kawachiensis* has been carried out by Igase and colleagues, who recorded improved cognitive function upon using AUR-enriched *C. kawachiensis* juice, and again, confirming neuroprotective effects of AUR^63^.

Given the wide-ranging scope of research and diverse studies, a compelling necessity emerges for a meticulous meta-analysis to join up the scattered knowledge on AUR and UMB. This analysis is pivotal not only for summarizing cumulative evidence but also for becoming a foundational reference guiding future clinical practice. Accordingly, the primary objective of this meta-analysis includes a comparative assessment of the cytotoxic effects of UMB and AUR across various cancer types. Moreover, this study aims to unveil the pivotal parameters determining the anticancer potential of AUR and UMB, and thus, provide crucial insights for their potential clinical applications.

## Methods

### Eligibility criteria

This meta-analysis aimed to evaluate the efficacy of AUR and UMB in cancer treatment, particularly by scrutinizing in vitro studies conducted on human cancer cells. To ensure the reliability and coherence of our analysis, we selectively incorporated original research articles providing crucial quantitative data. This encompassed vital details such as cell viability records at different concentrations or time intervals, along with IC_50_ values. Articles without this specific data in graphical representations or structured tabular formats were deliberately omitted to uphold the accuracy and robustness of our analysis. Moreover, to maintain uniformity in result interpretation, we exclusively focused on English-language publications.

### Information sources and search strategy

We conducted a systematic search across several databases, including PubMed, Lens, OA.mg, Google Scholar, and Cochrane Library, to identify studies focusing on the anticancer activity of AUR and UMB.

In PubMed, we utilized the following search terms: ("auraptene"[Title/Abstract]) AND ("cancer"[Title/Abstract]), ("umbelliprenin"[Title/Abstract]) AND ("cancer"[Title/Abstract]), (("natural"[Title/Abstract]) AND ("coumarin"[Title/Abstract])) AND ("cancer"[Title/Abstract]).

Within Lens, our search strategy incorporated the following terms: (title:("natural") OR abstract:("natural") OR keyword:("natural")) AND (title:("coumarin") OR abstract:("coumarin") OR keyword:("coumarin")) AND (title:("cancer") OR abstract:("cancer") OR keyword:("cancer")) AND (Title: ("auraptene") OR Abstract: ("auraptene") OR Keyword: ("auraptene")) AND (Title: ("umbelliprenin") OR Abstract: ("umbelliprenin") OR Keyword: ("umbelliprenin")) AND (Title: ("cancer") OR Abstract: ("cancer") OR Keyword: ("cancer")).

Moreover, we expanded our search beyond databases to OA.mg, Google Scholar and Cochrane Library. Our manual search involved the following terms: "auraptene" AND "anticancer activity" AND "human cells", "auraptene" AND "cancer inhibition" AND "in vitro", "auraptene" AND "cytotoxicity" AND "cancer cells", "umbelliprenin" AND "anticancer activity" AND "human cells", "umbelliprenin" AND "cancer inhibition" AND "in vitro", "umbelliprenin" AND "cytotoxicity" AND "cancer cells".

### Screening and data extraction

The articles extracted from the electronic database underwent preprocessing and screening using the "meta" tool^64^.

Initially, two independent investigators screened article titles and abstracts to determine eligibility. Subsequently, full-text versions of selected records were reviewed. Any reasons for excluding articles were documented, and any potential discrepancies were resolved through discussion until a consensus was achieved.

Considering the absence of a universally established or standardized scale for assessing bias in included in vitro studies, we adapted the widely-used Newcastle–Ottawa Scale (NOS)^65^. Originally designed for assessing the quality of non-randomized studies in meta-analyses, the NOS evaluates bias across three categories: Selection, Comparability, and Outcome.

The adaptation of the NOS was necessitated by the unique nature of in vitro studies, which significantly differ from non-randomized clinical trials or observational studies. In vitro studies involve diverse experimental conditions, cell lines, and quantitative outcome measures. To address these nuances, we tailored the NOS by emphasizing the quality and diversity of quantitative data reporting, as well as the inclusion of various experimental conditions. We aimed to ensure a comprehensive and rigorous evaluation of potential biases in the included in vitro studies. We tailored this scale to assess the risk of bias in the included in vitro studies as follows:

This adaptation enabled the evaluation of the risk of bias in overall studies.

- Selection: The chosen studies were required to provide essential quantitative data.
- Comparability: Evaluations were based on including diverse cell lines, concentrations, and time points in the studies. This diversity ensured that the studies were comparable and representative of the varying experimental conditions in vitro research.
- Outcome: Studies were assessed based on the reporting of outcomes as mean ± standard deviation (SD), presented in graphical formats with error bars or tabular layouts. This criterion ensured that the data reported in the studies were robust, statistically sound, and amenable to meta-analytical synthesis.

The extracted information from included articles encompassed various parameters, including the first author’s name and year of publication, cancer type, cell line used, details of the control group (type and dosage), treatment duration, and specifics about the coumarin type, its source, dosage, and unit.

Outcome measures included mean, SD, and the number of repeats in the treatment group, mean, SD, and the number of repeats in the control group, confidence intervals (CI), p-value, and the statistical method used for data analysis.

To note, concentrations initially not reported in µM units were converted to µM to ensure uniformity and maintain data integrity. For data presented in graphical formats, the extraction process was performed using WebPlotDigitizer^66^.

### Data visualization

To visually illustrate the distribution of data utilized in the synthesis, pie charts were employed, categorizing the information according to distinct cancer types. Additionally, a series of graphical representations were created to delve deeper into the relationships within the dataset. 3D scatter plots explored the interplay among coumarin concentration, time point, and viability, aiming to discern underlying trends. Meanwhile, 2D scatter plots were constructed to depict the relationship between coumarin dose and viability, with data points categorized by cancer type for enhanced clarity and analysis. Another 2D scatter plot depicting coumarin dose against viability, augmented by fitted regression lines, was also generated to elucidate trends and variations across different cell lines.

In addition to individual scatter plots, heat maps illustrating correlations among coumarin dose, viability, and time were generated. Heatmaps served to unearth potential associations between variables and cell viability across various coumarin types. All graphical representations were crafted utilizing the matplotlib library.

### Machine learning

Building upon these visualizations, a Machine Learning (ML) approach was employed to identify the most influential features contributing to the viability percentage in different cancer types based on selected parameters. The data were loaded using the Pandas library. Categorical variables, such as 'cancerType' and 'coumarin,' were encoded using the LabelEncoder from Scikit-Learn to convert them into a numerical format for ML modeling. The 'viability' values were utilized to classify instances into two groups: 'Highly responsive' (1) when viability was below the median. 'Lowly responsive' (0) when viability was above or equal to the median. The threshold for responsiveness ('viability_threshold') was set at the median viability. Features ('time', 'coumarinDose', 'cancerType', 'coumarin') were selected to predict the 'response' (highly/lowly responsive). The data were divided into training and testing sets using the train_test_split method from Scikit-Learn with an 80:20 split ratio and a fixed random seed. A Random Forest Classifier from Scikit-Learn was instantiated and trained using the training data (X_train, y_train). The trained model was used to predict responsiveness on the test set (X_test), and predictions ('y_pred') were obtained. The model's performance was assessed using accuracy and a detailed classification report (precision, recall, F1-score) generated via the classification_report function from Scikit-Learn. Feature importance was calculated using the RandomForestClassifier. The top contributing features were visualized using a bar plot to display their importance scores. The Seaborn library was used to create a heatmap visualizing precision, recall, and F1-score values for various classes.

The Random Forest Classifier was chosen due to its ability to handle both continuous and categorical variables, as well as its interpretability in terms of feature importance. However, it is important to note that the primary objective of this ML analysis was to quantify the relative contributions of the examined features in determining the anticancer effects of AUR and UMB, rather than develop a highly accurate predictive model. Extensive hyperparameter tuning was not performed due to data limitations.

### Data synthesis

The synthesis of our data involved separate analyses for AUR and UMB, utilizing the standardized mean difference as the outcome measure, with coumarin dose as a moderator. To process the data, a random-effects model was employed. This included estimating the amount of heterogeneity (tau^2^) via the restricted maximum-likelihood estimator^67^. Additionally, we reported the Q-test for heterogeneity^68^ and the I^2^ statistic. When any level of heterogeneity was detected (tau^2^ > 0), irrespective of the Q-test results, a prediction interval for true outcomes was provided. The computations, tests, and confidence intervals were obtained using the Knapp and Hartung method^69^. In handling missing SDs for each data entry, our approach involved computing the median or mean of other available SDs within the same study. When no SD was mentioned for the control group, we applied a default value 1.

For assessing publication bias, we employed visual funnel plots and Fail-Safe N calculations. Sensitivity analysis was conducted by progressively removing individual studies to gauge their impact on the results. All analyses were performed using Jamovi version 2.4, with statistical significance set at *p* < 0.05 for two-tailed tests.

## Results

**Figure 2:**
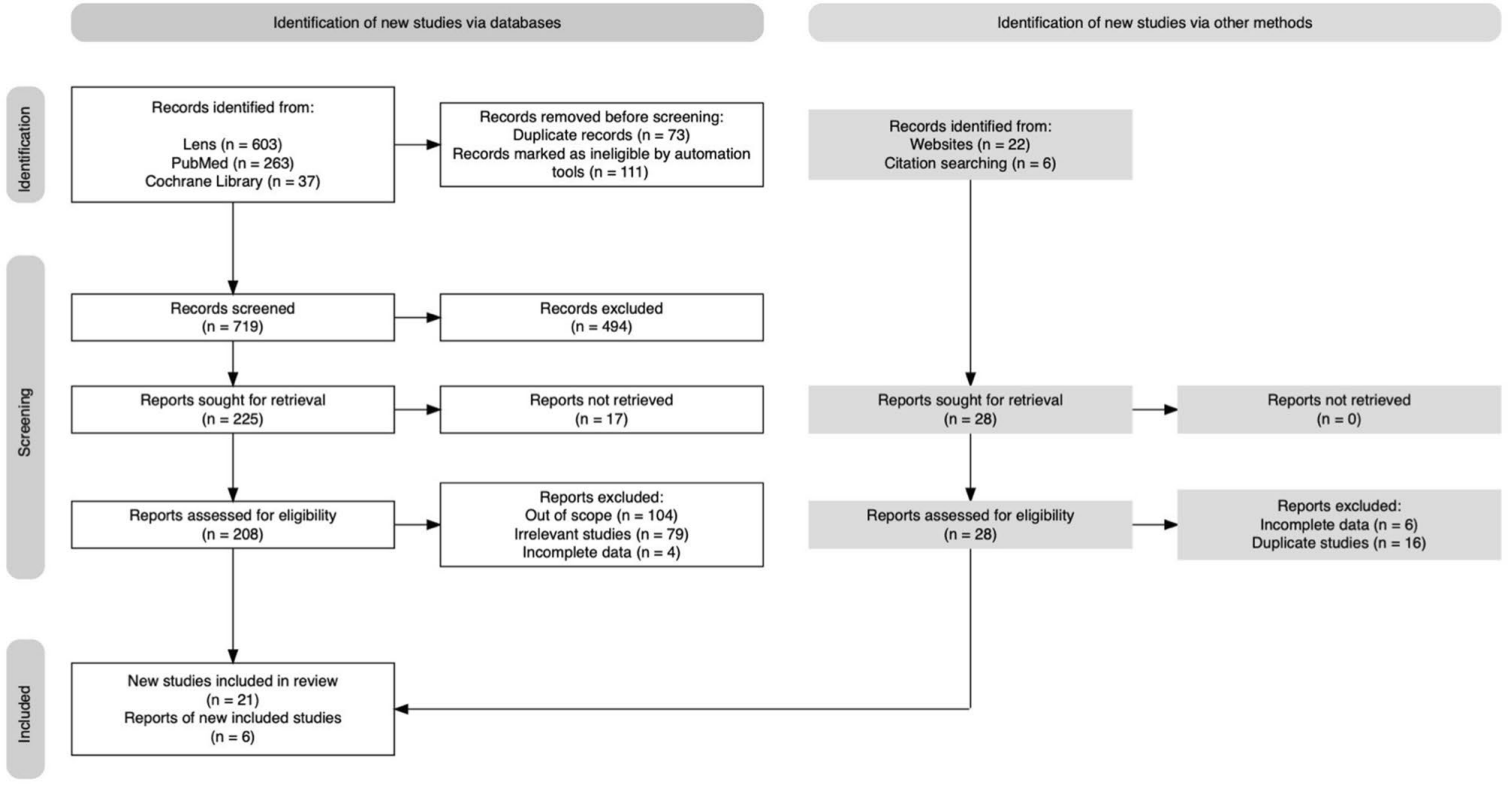
PRISMA flow diagram illustrating the study selection process.

The database search initially yielded 903 publications (Fig. 2). After the initial screening, 73 duplicates were identified, and an additional 111 were deemed ineligible by automation tools. Subsequently, 494 publications were excluded based on title and abstract screening, and after a full-text review of the remaining 225 articles, 21 articles met the criteria for inclusion in the meta-analysis. In parallel, the manual search identified 28 publications, all of which underwent full-text review, and finally, 6 were considered suitable for inclusion in the meta-analysis. A summary of the included publications' key data is presented in Table 1^8,19,23,27,30,33,34,40,41,46,47,49,50,56,70–80^.

**Table 1:** Characteristics of included studies.

Studies	Coumarin	Cancer	Cell line	Dose (µM)	Time (h)	Outcome *	Risk of bias **
Abolhassani et al. 2023	AUR	Prostate	PC3	5–80	48 72 96 120 144	treatment (n = 3): 1) 86.98 ± 6.82 2) 79.37 ± 5.52 3) 51.37 ± 5.33 control (n = 3): 84.7 ± 6.66	Selection: ♦♦ Comparability: ♦♦♦♦♦ Outcome: ♦♦
Afshari et al. 2019	AUR	Glioblastoma	U87	167–335	8 24	treatment (n = 3): 6) 64.04 ± 3.05 7) 47.22 ± 3.11 control (n = 3): 100 ± 1.87	Selection: ♦♦ Comparability: ♦♦♦♦ Outcome: ♦♦
Bagheri et al. 2022	AUR	Leukemia/Lymphoma	MT-2	16.7–134	24 48 72 96 120 144	treatment (n = 3): 2) 92.36 ± 5.07 3) 74.84 ± 5.57 control (n = 3): 95.96 ± 4.45	Selection: ♦♦ Comparability: ♦♦♦♦ Outcome: ♦♦
Charmforoshan et al. 2019	AUR	Breast	MCF-7	61–207	48	treatment (n = 3): 3) 50 ± 3.65 4) 75 ± 4.79 7) 49.24 ± 2.52 control (n = 3): 100 ± 3.36	Selection: ♦♦ Comparability: ♦♦♦ Outcome: ♦♦
Epifano et al. 2013	AUR	Colon	HT-29 HT-116	2.5–40	72	treatment (n = 3): 1) 82.32 ± 10.88 2) 46.41 ± 10 control (n = 3): 99.81 ± 10.75	Selection: ♦♦ Comparability: ♦♦♦♦♦ Outcome: ♦♦
Epifano et al. 2023	AUR	Neuroblastoma	SHSY-5Y	12.3–50	24	treatment (n = 6): 2) 78.39 ± 5.82 3) 62.04 ± 5.81 control (n = 6): 100 ± 3.36	Selection: ♦♦ Comparability: ♦♦♦ Outcome: ♦♦
Gkionis et al. 2021	AUR	Breast	MCF-7 MDA-MB231	1–100	48	treatment (n = 2): 1) 97.43 ± 8.7 3) 56.97 ± 6.13 4) 26.11 ± 6.82 control (n = 2): 100 ± 3.36	Selection: ♦♦ Comparability: ♦♦♦ Outcome: ♦♦
Goudarzi et al. 2022	AUR	Leukemia/Lymphoma	MT-2	10–40	48 72 96	treatment (n = 3): 1) 92.54 ± 6.38 2) 87.64 ± 6.89 control (n = 3): 99.4 ± 1.76	Selection: ♦♦ Comparability: ♦♦♦♦ Outcome: ♦♦
Izadi et al. 2023	AUR	Glioblastoma	U87	40–330	24	treatment (n = 3): 2) 89.38 ± 11.02 3) 80.81 ± 2.04 7) 46.53 ± 2.45 control (n = 3): 100 ± 3.36	Selection: ♦♦ Comparability: ♦♦♦ Outcome: ♦♦
Jalilzade et al. 2020	AUR	Colon	HT-29	12.5–100	24 48 72	treatment (n = 3): 2) 81.51 ± 3.42 3) 60.86 ± 3.47 4) 52.23 ± 3.22 control (n = 3): 99.91 ± 2.13	Selection: ♦♦ Comparability: ♦♦♦♦ Outcome: ♦♦
Jamialahmadi et al. 2018	AUR	Ovarian Cervical	A2780 HeLa	3–100	24	treatment (n = 3): 1) 86.12 ± 6.95 2) 63.56 ± 8.47 3) 51.96 ± 6.18 control (n = 3): 100 ± 3.36	Selection: ♦♦ Comparability: ♦♦♦♦♦ Outcome: ♦♦
Krishnan et al. 2009	AUR	Breast	MDA-MB231 MCF-7	1–50	24	treatment (n = 3): 1) 95.41 ± 7.34 3) 34.67 ± 3.94 control (n = 3): 100 ± 6.19	Selection: ♦♦ Comparability: ♦♦♦♦♦ Outcome: ♦♦
Lee et al. 2017	AUR	Prostate	PC3 LNCaP DU145	15–120	24	treatment (n = 3): 2) 79.91 ± 3.49 3) 63.39 ± 2.42 4) 49.73 ± 3.05 control (n = 3): 100 ± 2.8	Selection: ♦♦ Comparability: ♦♦♦♦♦ Outcome: ♦♦
Li et al. 2013	AUR	Breast	T47D MDA-MB231	1–30	48 144	treatment (n = 3): 1) 87.19 ± 2.88 control (n = 3): 100 ± 3.36	Selection: ♦♦ Comparability: ♦♦♦♦♦ Outcome: ♦♦
Moon et al. 2015	AUR	Gastric	AGS MKN-45 SNU-1 SNU-16	12.5–100	48	treatment (n = 3): 2) 84.26 ± 11.51 3) 48.53 ± 6.51 4) 37.89 ± 3.3 control (n = 3): 100 ± 5.31	Selection: ♦♦ Comparability: ♦♦♦♦ Outcome: ♦♦
Movaffagh et al. 2023	AUR	Gastric	MKN-45	16.7–133.6	24 48 72 96 120	treatment (n = 3): 2) 84.32 ± 5.72 3) 65.43 ± 7.92 5) 43.63 ± 6.38 control (n = 3): 91.8 ± 3.5	Selection: ♦♦ Comparability: ♦♦♦♦ Outcome: ♦♦
Shiran et al. 2021	AUR	Breast	MDA-MB231	6.25–200	24	treatment (n = 3): 1) 94.44 ± 2.96 2) 89.81 ± 2.41 3) 84.07 ± 2.59 4) 58.14 ± 2.6 control (n = 3): 100 ± 3.36	Selection: ♦♦ Comparability: ♦♦♦ Outcome: ♦♦
Barthomeuf et al. 2008	UMB	Colon Breast Ovarian Melanoma Prostate Lung	DLD1 MCF-7 PA1 M4Beu PC3 A549	27.3	48	treatment (n = 3): 2) 45.24 ± 3.27 control (n = 3): 100 ± 2.29	Selection: ♦♦ Comparability: ♦♦♦♦ Outcome: ♦♦
Gkionis et al. 2021	UMB	Breast	MCF-7 MDA-MB231	1–100	48	treatment (n = 2): 1) 96.98 ± 8.1 4) 46.93 ± 4.99 control (n = 2): 100 ± 2.29	Selection: ♦♦ Comparability: ♦♦♦ Outcome: ♦♦
Goodarzi et al. 2022	UMB	Leukemia/Lymphoma	MT-2	1–80	48 72 96	treatment (n = 3): 3) 65.01 ± 7.06 control (n = 3): 99.36 ± 1.96	Selection: ♦♦ Comparability: ♦♦♦♦ Outcome: ♦♦
Hamidinia et al. 2013	UMB	Colon	SW48	6.25–200	24 48 72	treatment (n = 3): 1) 90.83 ± 2.17 2) 83.22 ± 1.69 3) 67.03 ± 3.74 4) 42.4 ± 2.05 6) 11.66 ± 1.51 control (n = 3): 100 ± 2.29	Selection: ♦♦ Comparability: ♦♦♦♦♦ Outcome: ♦♦
Kamalkazemi et al. 2023	UMB	Colon	HT-29	20–140	24 48	treatment (n = 3): 2) 64.34,1.95 3) 42.77 ± 1.28 4) 28.57 ± 1.46 5) 16.82 ± 1.88 control (n = 3): 100 ± 2.29	Selection: ♦♦ Comparability: ♦♦♦♦ Outcome: ♦♦
Khaghanzadeh et al. 2012	UMB	Lung	A549 QU-DB	10–200	24 48 72	treatment (n = 3): 1) 89.63 ± 2.57 2) 82 ± 4.15 3) 68.37 ± 4.73 4) 33.91 ± 7.72 6) 13 ± 3 control (n = 3): 94.16 ± 2.03	Selection: ♦♦ Comparability: ♦♦♦♦♦♦ Outcome: ♦♦
Rashidi et al. 2016	UMB	Colon Breast Glioma	HT-29 MCF-7 A172	8.1–272.8	24 48 72	treatment (n = 3): 1) 98.88 ± 6.22 2) 84.7 ± 4.88 3) 64.43 ± 5.15 5) 36.6 ± 3.82 7) 16.85 ± 2.62 control (n = 3): 100 ± 2.29	Selection: ♦♦ Comparability: ♦♦♦♦♦♦ Outcome: ♦♦
Zhang et al. 2019	UMB	Gastric	AGS BGC-823	3.13–50	24 48 72	treatment (n = 3): 1) 71.12 ± 3.44 2) 40.11 ± 3.98 3) 16.82 ± 2.92 control (n = 3): 99.63 ± 1.94	Selection: ♦♦ Comparability: ♦♦♦♦♦ Outcome: ♦♦
Zhang et al. 2015	UMB	Gastric Cervical Lung Prostate	AGS HeLa A549 PC3	6.25–121	24	treatment (n = 3): 1) 87.72 ± 1.43 2) 67.43 ± 2.38 3) 49 ± 4.71 4) 35 ± 2.14 5) 50 + 2.28 control (n = 3): 1) 97.72 ± 1.72 2,3,4) 98.48 ± 1.72 5) 100 ± 1.72	Selection: ♦♦ Comparability: ♦♦♦♦ Outcome: ♦♦
Ziai et al. 2012	UMB	Leukemia	Jurkat T	10–100	16 48	treatment (n = 3): 1) 80.83 ± 2.34 2) 68.25 ± 3.54 3) 44.38 ± 3 4) 26.72 ± 2.93 control (n = 3): 100 ± 2.29	Selection: ♦♦ Comparability: ♦♦♦♦ Outcome: ♦♦

*Viability ± SD, subgroups = {1: ≤ 16.7 µM, 2: 16.7–40, 3: 40–80 µM, 4: 80–120 µM, 5: 120–160 µM, 6: 160–200 µM, 7: > 200 µM}.

**Maximum score: ♦♦ for selection, ♦♦♦♦♦♦ for comparability, and ♦♦ for outcome.

**Figure 3:**
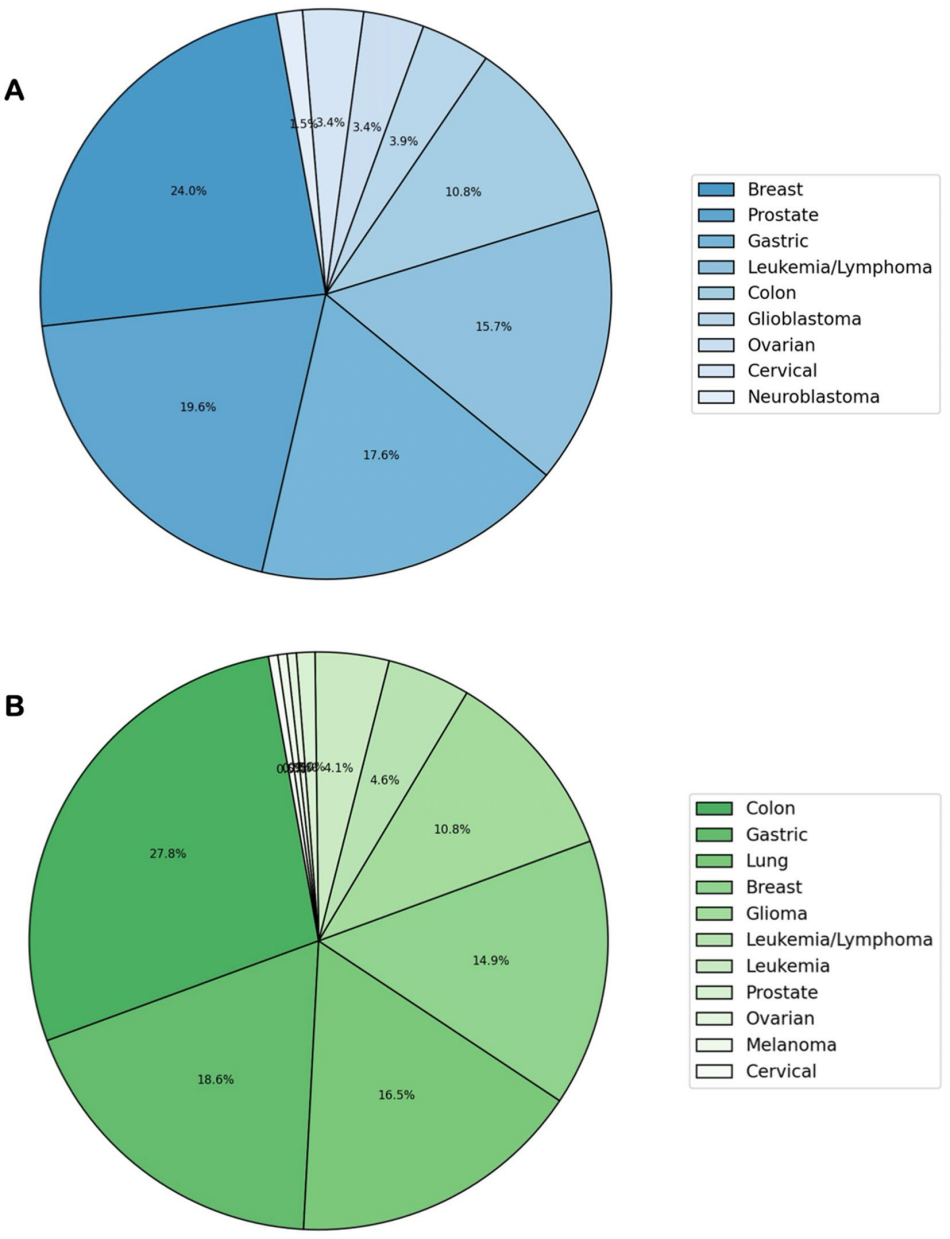
Pie charts illustrating the distribution of cancer types in AUR (**A**) and UMB (**B**) datasets.

In Fig. 3, pie charts illustrate the distribution of collected data on different cancer types for AUR and UMB. For AUR, the dataset distribution depicts the highest representation of breast cancer, constituting 24.0% of the data. This is followed by prostate cancer at 19.6%, gastric cancer at 17.6%, leukemia/lymphoma at 15.7%, and colon cancer at 15.0%. The remaining cancer types comprise smaller proportions, ranging from 1.5% to 10.8%. Similarly, the most significant dataset for UMB corresponds to colon cancer, contributing 27.8% of the data. This is succeeded by gastric cancer at 18.6%, lung cancer at 16.5%, breast cancer at 14.9%, and colon cancer at 10.8%. The remaining cancer types encompass smaller percentages, ranging from 0.05% to 4.6%.

**Figure 4:**
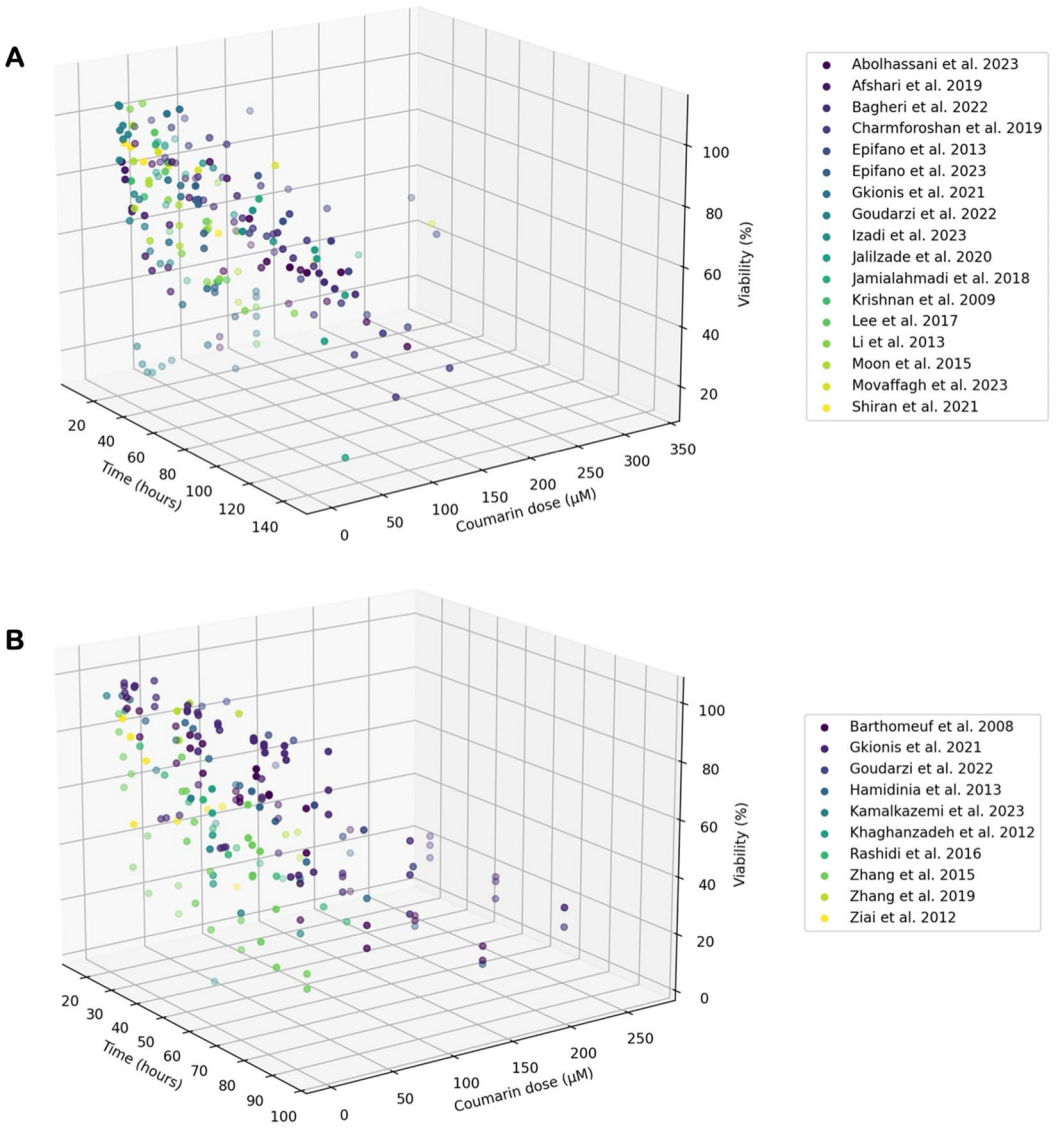
3D scatter plots illustrating the relationship among time (h), coumarin dose (µM), and viability (%) for AUR (**A**) and UMB (**B**) in different studies. Each data point is color-coded, denoting individual studies.

Figure 4 represents 3D scatter plots that portray the interplay among time, coumarin dose, and viability. Here, the x-axis signifies time (h), the y-axis represents coumarin dose (µM), and the z-axis indicates viability (%). The 3D plot of AUR demonstrates a consistent negative correlation between coumarin dose and viability across all studies. This universal trend underscores the consistent impact of AUR on reducing cell viability, irrespective of study variations. Likewise, the 3D plot of UMB reveals a negative correlation between coumarin dose and viability and indicates the potential of UMB as an anticancer agent. To note, considerable variations among study results are apparent, potentially stemming from diverse experimental methods, cell types utilized, or other influencing factors.

**Figure 5:**
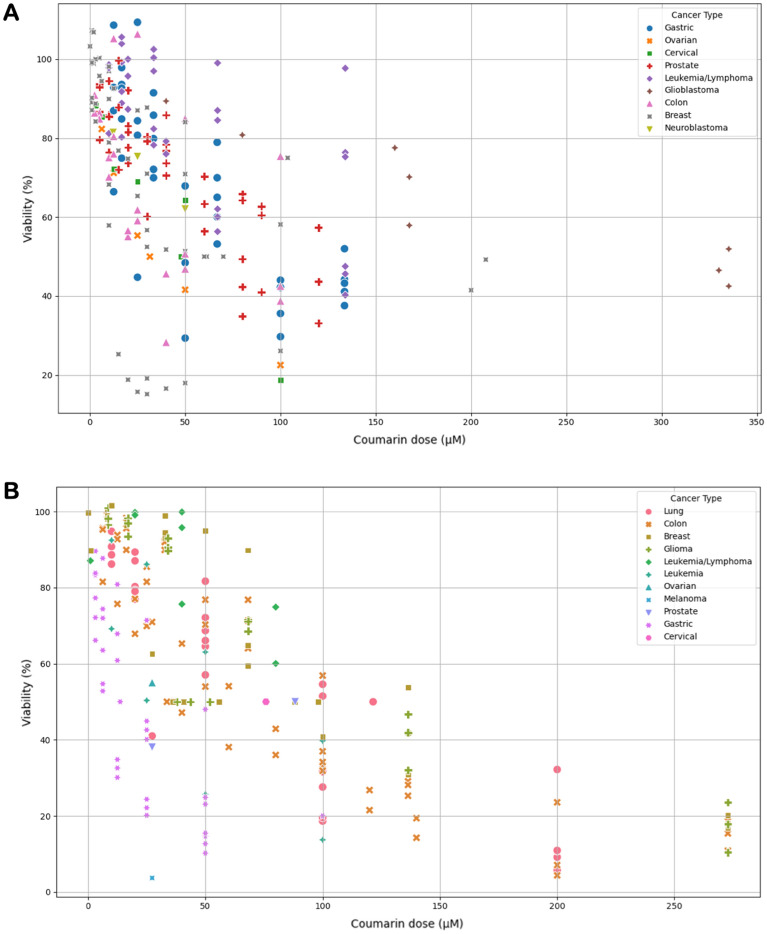
2D scatter plots illustrating the correlation between coumarin dose (µM) and viability (%) for AUR (**A**) and UMB (**B**) across various cancer types. Each cancer type is distinguished by a unique color and symbol in the data representation.

Examining the relationship between coumarin dose and cancer type in 2D scatter plots reveals a negative correlation for AUR (Fig. 5A). This indicates that with an increase in coumarin dose, there is a corresponding decrease in viability. Notably, the data related to gastric cancer slightly deviates from other cancer types, hinting at potentially higher sensitivity to AUR compared to the rest. Similarly, in the case of UMB (Fig. 5B), a negative correlation is observed between coumarin dose and viability. This relationship seems consistent across all cancer types, although the extent of the impact varies among different types. This suggests that while the influence of UMB on cell viability remains uniform across the board, the degree of this effect fluctuates across distinct cancer types.

**Figure 6:**
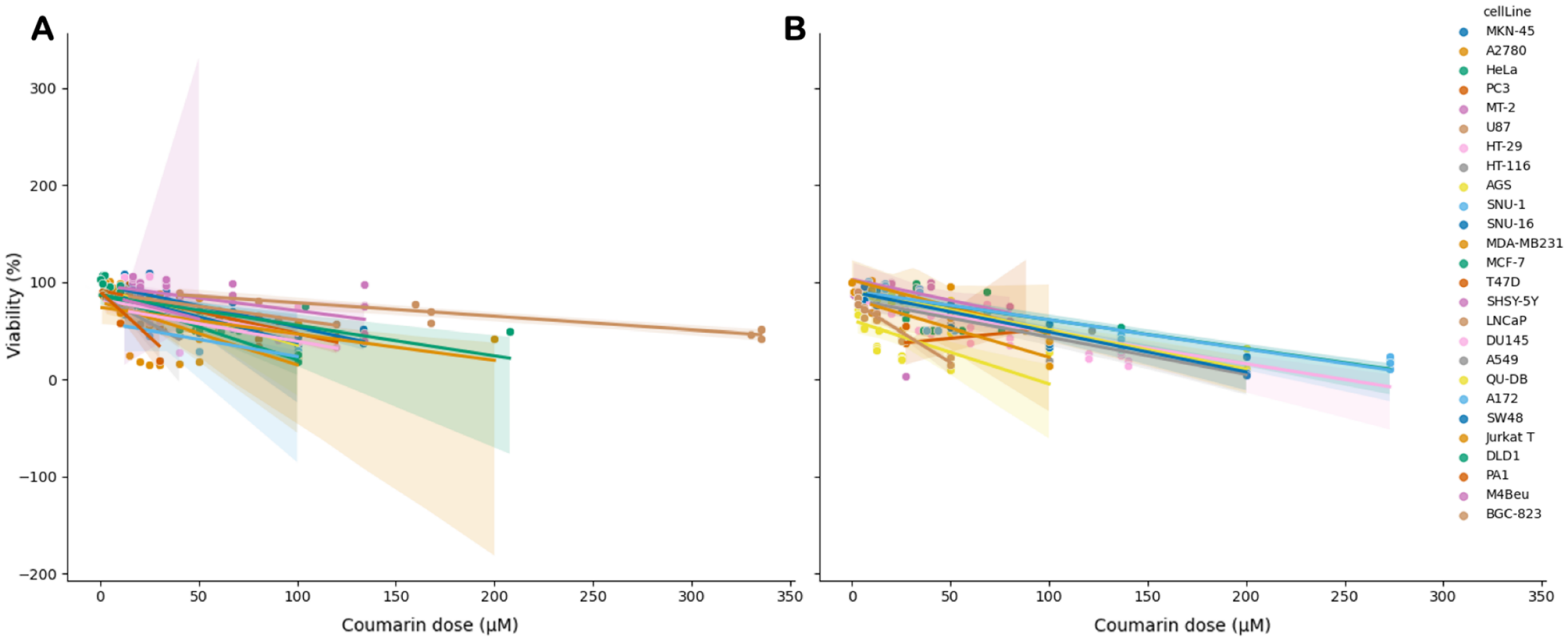
The relationship between coumarin dose (µM) and viability (%) in different cell lines for AUR (**A**) and UMB (**B**). Each cell line is distinguished by a unique color in the data representation.

The relationship between coumarin dose and viability in different cell lines is also depicted in Fig. 6. Although variations between cell lines and the pattern is complex, higher doses tend to be associated with lower viability.

**Figure 7:**
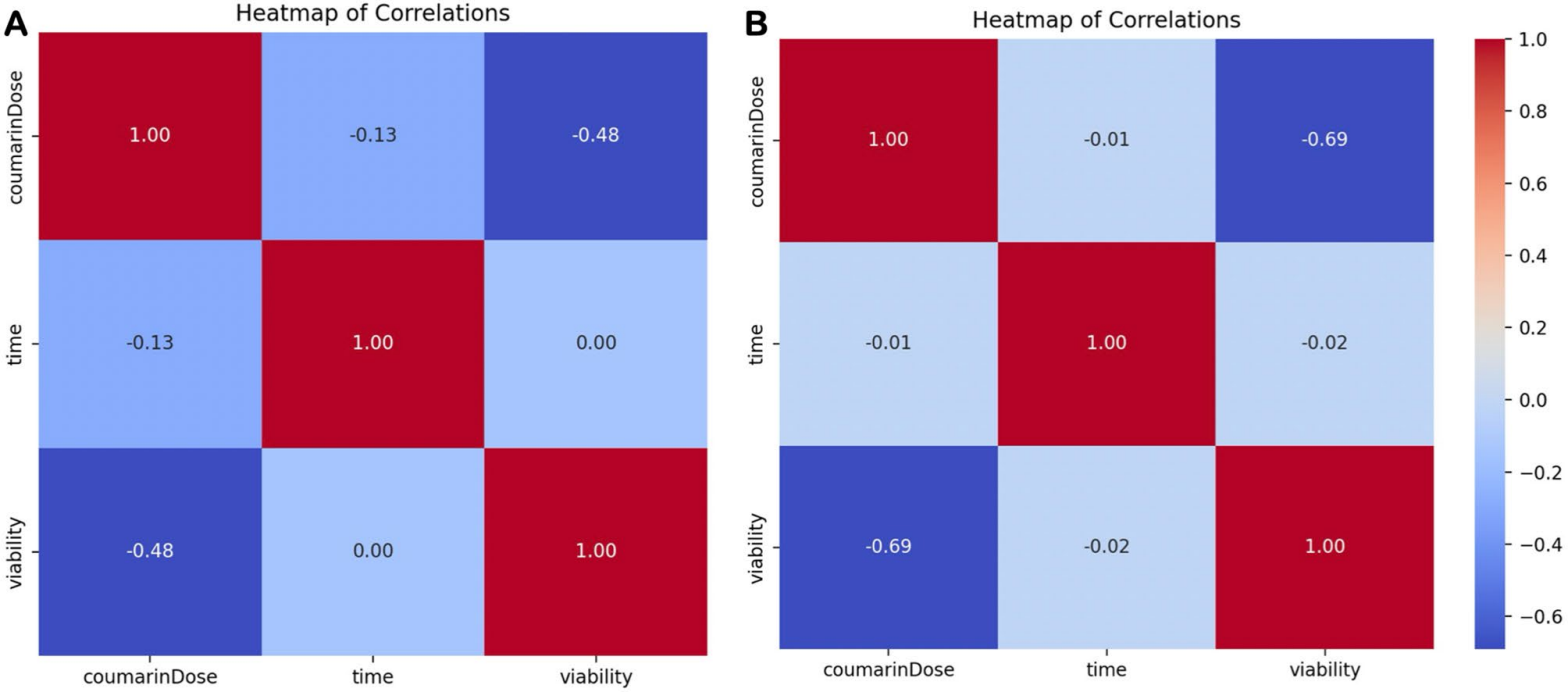
Heatmaps of correlations between coumarin dose, time, and viability for AUR (**A**) and UMB (**B**).

The heatmap of correlations for AUR (Fig. 7A) demonstrates a robust negative correlation (− 0.48) between coumarin dose and viability, in line with the previously displayed scatter plots. In contrast, the correlation between time and viability is notably weak (0.00), suggesting a lack of significant association between these variables. The heatmap of correlations for UMB (Fig. 7B) reveals a stronger negative correlation (− 0.69) between coumarin dose and viability compared to AUR. This reiterates the earlier trend, indicating a more pronounced decrease in viability with escalating coumarin doses. Similiar to AUR, the correlation between time and viability remains insignificantly weak (− 0.02), indicating no substantial relationship between these variables for UMB.

**Figure 8:**
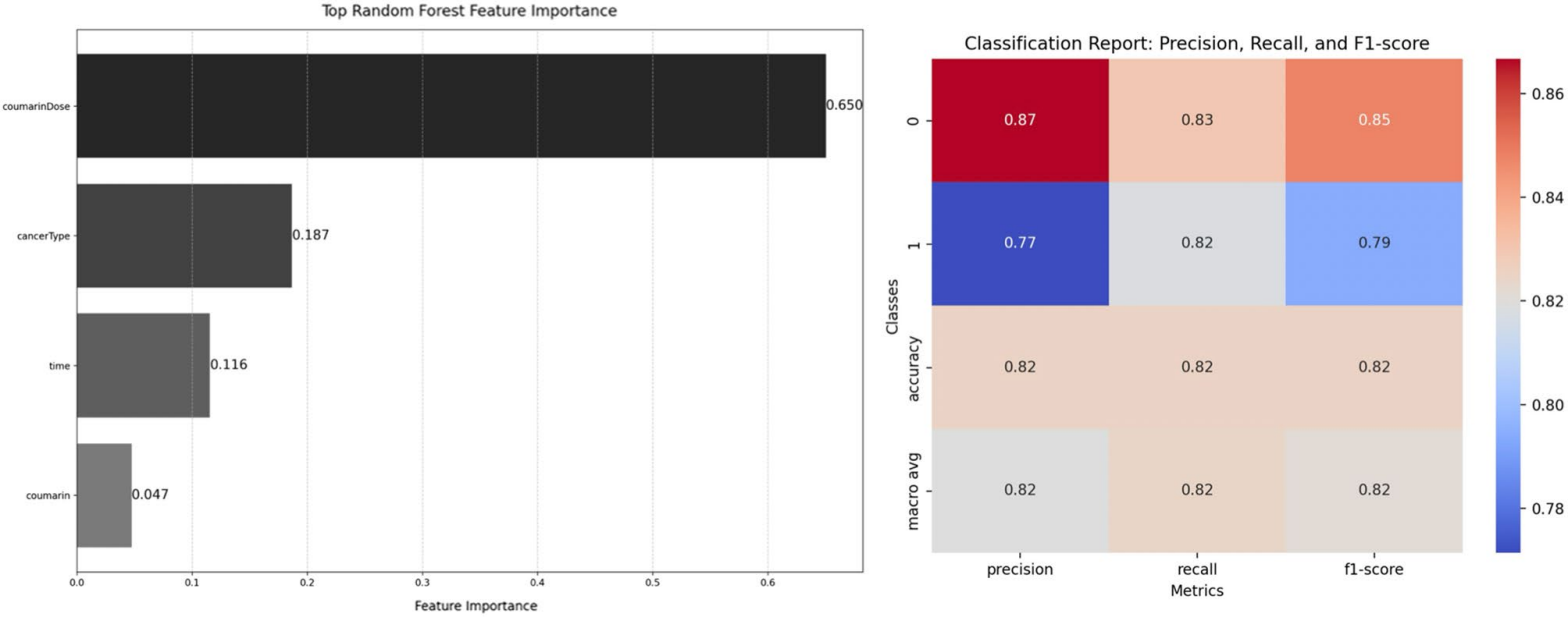
RandomForestClassifier-based feature importance plot highlighting factors influencing the anticancer potential of coumarins (left). The classification report of the RandomForestClassifier model predicting cancer cell viability using multiple parameters (right).

The RandomForestClassifier-derived feature importance (Fig. 8) sheds light on various parameters impacting the prediction of coumarin toxicity. Foremost among variables is the coumarin dose, contributing significantly with over 65% accuracy in the model that highlights the pivotal role of dose in determining the anticancer potential. Additionally, cancer type and treatment duration emerge as significant factors, contributing approximately 19% and 12% to the model's accuracy, respectively. In contrast, the nature of coumarin, whether AUR or UMB, demonstrates a relatively lower impact, contributing only about 5% to the model's accuracy. This suggests that while distinguishing between AUR and UMB is pertinent, their chemical similarities might result in analogous behaviors across various cancer types and cell lines. Thus, variables beyond the nature of coumarin—such as dose—primarily define the anticancer potential of both agents and underscore their parallel behavior across different cancer types. The classification report of the RandomForestClassifier model predicts cancer cell viability using multiple parameters. The model demonstrated promising capabilities in forecasting cancer cell viability, achieving an 82% overall accuracy in correctly classifying test data based on coumarin type and dose, cancer type, and treatment time (Fig. 8).

A total of 27 studies were incorporated into the analysis, investigating the anticancer effects of either AUR or UMB across a spectrum of cancer cell lines. The administered doses ranged from 1 to 335 μM, while treatment durations varied from 16 to 144 h. The reported outcomes primarily focused on cell viability percentages compared to untreated control groups. Of the total studies included, 78% (21 out of 27) were determined to have a low risk of bias. Elevated risk of bias was presumably attributed to a lack of diversity in cell lines, concentrations, and time points within some studies, impacting the overall assessment of these studies.

**Figure 9:**
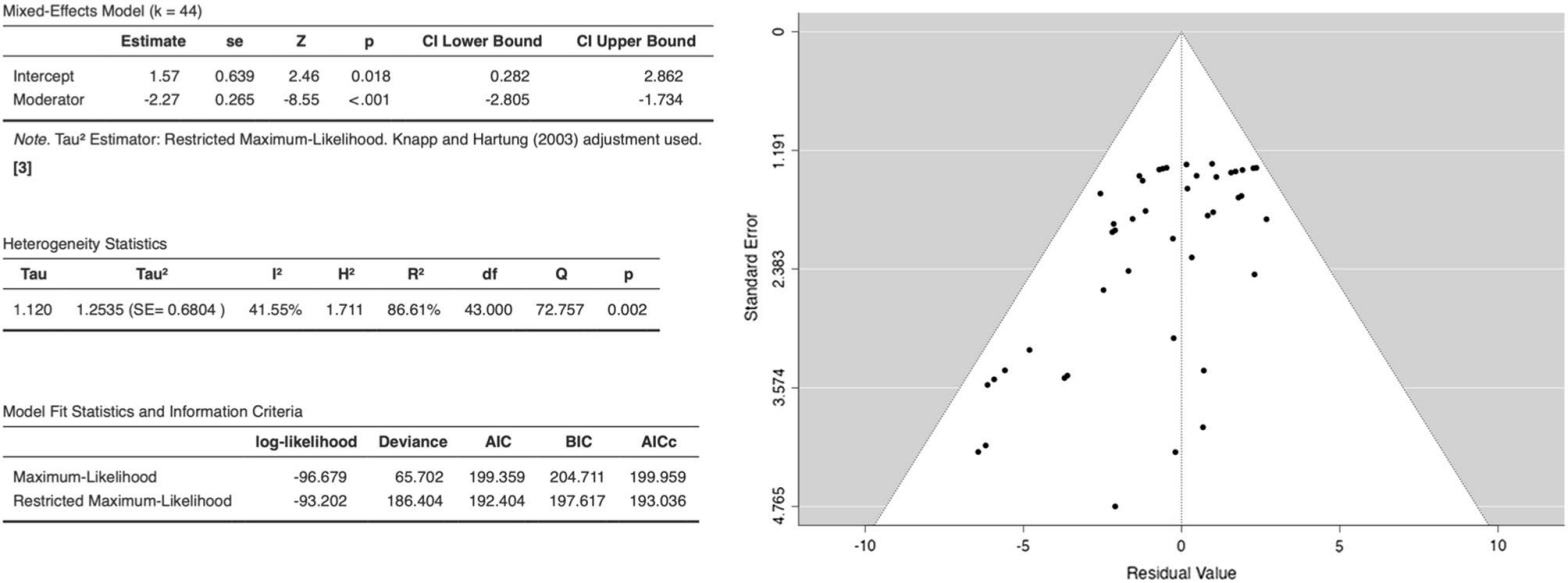
Meta-analysis of AUR: summary of statistics (left) and funnel plot (right).

**Figure 10:**
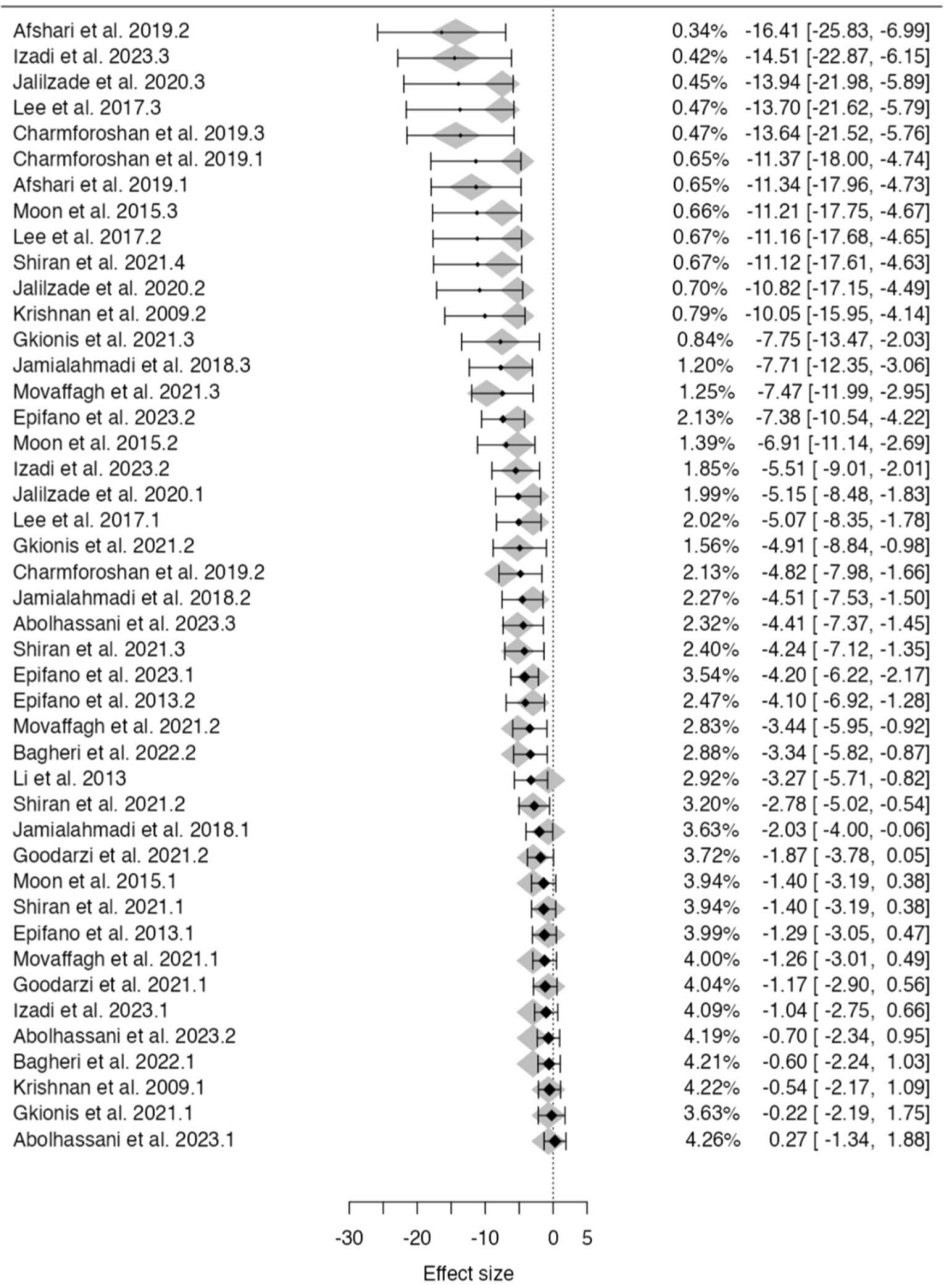
Forest plot meta-analysis of anticancer effects of AUR across 17 studies with coumarin dose as a moderator. Study weights and estimated effect sizes with 95% confidence intervals are shown.

Meta-analysis was conducted examining the effects of AUR on cancer cell viability across 17 studies (Fig. 9). A mixed-effects model revealed a significant negative association between AUR dose and cancer cell viability (est. = − 2.27, 95% CI [− 2.805, − 1.734], z = − 8.55, p < 0.001), indicating AUR acts in a dose-dependent manner. Figure 10 represents the forest plot for the anticancer effects of AUR. Moderate between-study heterogeneity was observed (I2 = 41.55%, p = 0.002), suggesting variable effects beyond sampling error.

Publication bias assessment indicated potential issues with minor study effects, with Begg and Egger's tests rejecting null hypotheses (p < 0.001). However, the high and statistically significant fail-safe N value of 4631 suggests overall results are robust to these biases. According to Cook’s distances, none of the studies could be considered overly influential.

Overall, although some publication bias is possible, the meta-analysis provides consistent evidence across 17 studies that AUR significantly reduces cancer cell viability in a dose-dependent manner. Further examination of heterogeneity sources is warranted, but effects appear robust and sizable at higher AUR doses.

**Figure 11:**
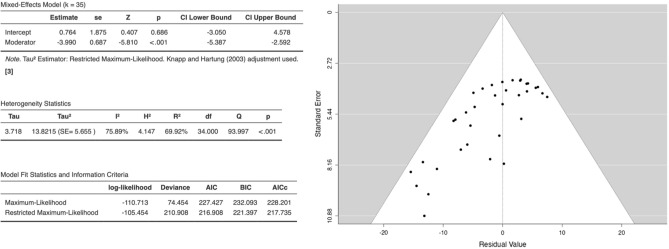
Meta-analysis of UMB: summary of statistics (left) and funnel plot (right).

**Figure 12:**
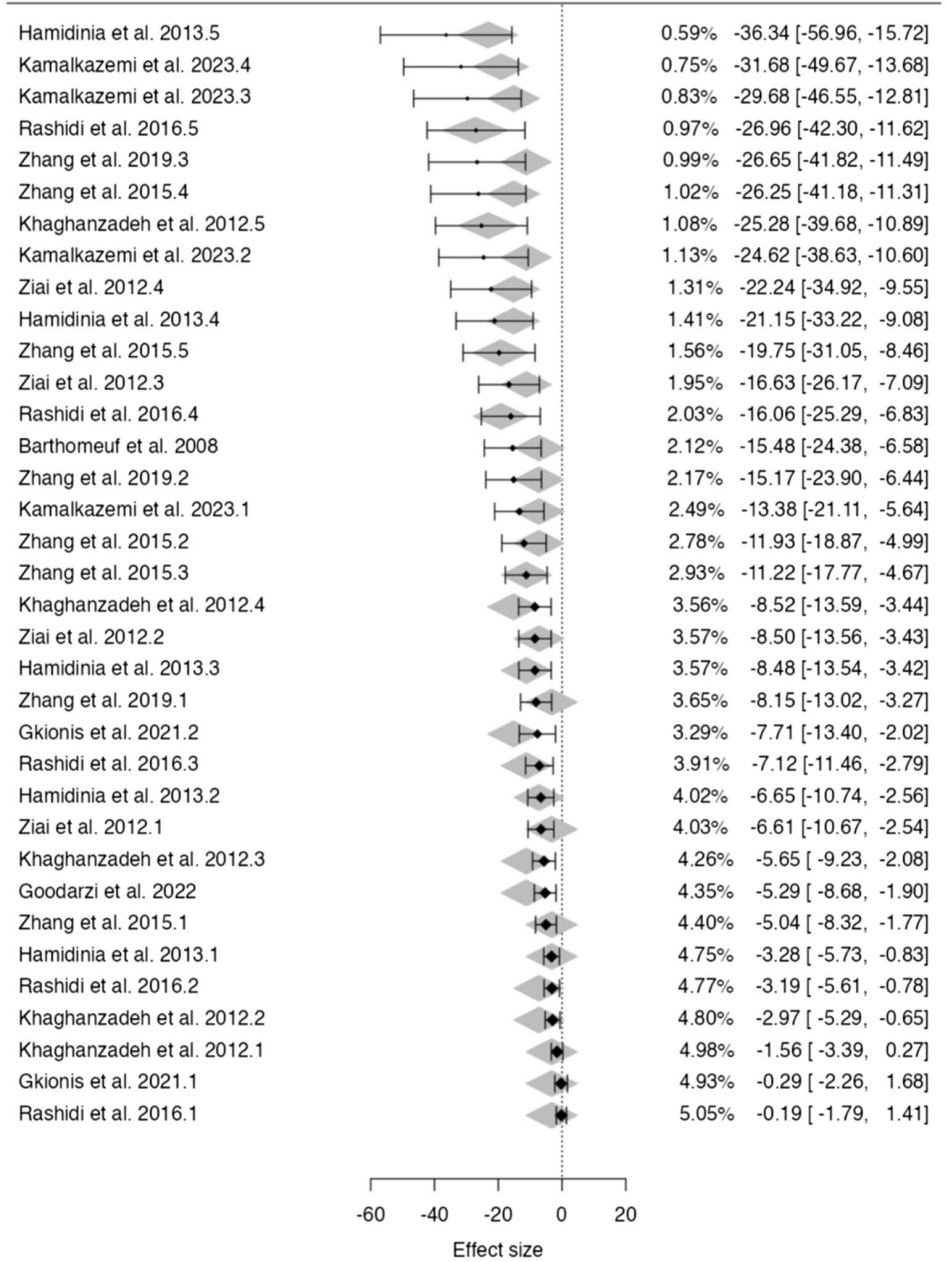
Forest plot meta-analysis of anticancer effects of UMB across 10 studies with coumarin dose as a moderator. Study weights and estimated effect sizes with 95% confidence intervals are shown.

Figure 11 depicts the results of a mixed-effects meta-analysis that was conducted to assess the efficacy of UMB on cancer cell lines. Effects were analyzed across 10 studies, with coumarin dose examined as a moderator. A random effects model was utilized, given anticipated heterogeneity. Results demonstrated no significant overall effect of UMB based on the intercept (est. = 0.764, 95% CI [− 3.05, 4.58], z = 0.407, p = 0.686). However, the effect of the moderator was significant (est. =  − 3.990, 95% CI [− 5.39, − 2.59], z = − 5.810, p < 0.001), suggesting viability decreases as the dose increases. Figure 12 is the forest plot for the anticancer effects of UMB. Significant study heterogeneity was present (I2 = 75.89%, p < 0.001). Outlier diagnostics showed no externally standardized residuals exceeding critical values. There was evidence of publication bias based on Begg and Egger’s tests. However, the considerable and statistically significant fail-safe N value of 4126 indicates a high level of robustness in the overall findings, even in the presence of these biases.

To sum up, while no overall effect emerged for UMB efficacy across cancer cell lines, coumarin dose evidenced a negative moderating effect on efficacy. Possible publication bias may be present according to statistical tests, and model comparisons suggest this data may be overfitted by maximum likelihood estimation. Further trials appear warranted, especially investigating efficacy by concentration.

## Discussion

Failure in current anticancer modalities has posed challenges to the biomedical community for developing effective alternatives. The limited curative efficacy, elevated toxicity, and lack of selectivity are vital constraints that restrict the utilization of current chemotherapy regimens. This emphasizes the critical requirement for innovative anticancer agents to alleviate patient suffering and mitigate the high costs associated with current treatments^5^. Plant-derived agents have attracted much attention due to their valuable pharmaceutical activities, with a notable interest in natural coumarins, which exhibit lower side effects on normal cells and higher efficacy towards cancer cells. In the present study, we aimed to systematically assess, compare, and quantify the anticancer efficacy of AUR and UMB by synthesizing evidence from 27 studies. The results demonstrate a consistent negative correlation between coumarin dose and cancer cell viability across studies, indicating both AUR and UMB induce their anticancer effects in a dose-dependent manner.

Employing an ML approach indicated that among the parameters analyzed, coumarin dose emerged as the most critical feature determining toxicity, followed by cancer type and treatment duration. However, the nature of the coumarin (AUR or UMB) demonstrated minimal impact, presumably because structural similarities result in analogous behaviors.

Meta-analysis revealed moderate and substantial between-study heterogeneities for AUR and UMB, respectively. Potential sources may include variations in the experimental methods, cell lines, or other parameters across studies. Nonetheless, the sizable fail-safe N values indicate considerable robustness of findings for both coumarins despite heterogeneity.

Although no overall UMB effect emerged, its moderator, coumarin dose, evidenced a solid negative association (est. = − 3.990) with viability, while for AUR, a moderate estimated effect size (est. = − 2.27) was demonstrated. The more potent impact of UMB dose may arise from its longer aliphatic farnesyl chain that can enhance lipophilicity and biological activity compared to the geranyl chain in the AUR chemical structure. Gkionis and colleagues have reported superior bioactivity of UMB over AUR, aligning with our analysis^8^.

The minor contribution of the coumarin type highlights similarities between AUR and UMB, potentially due to overlapping structural features imparting analogous behavior. However, as mentioned earlier, additional prenyl units in the UMB structure might slightly enhance anticancer effects by improving access, affinity, and interaction with the lipophilic cellular membranes^7^. Further structure–activity studies focusing on the substitutions and side chains are recommended.

Despite the current meta-analysis providing valuable insights into the anticancer efficacy of AUR and UMB, some limitations persist. Firstly, the viability predictions apply solely to the cancer cell lines examined, with caution needed in extrapolating to other cancers lacking representation in the dataset. Also, one of the critical gaps in the current literature, which our study also encounters, is the inconsistency in the dose ranges used across different studies. Some studies report non-standard dose ranges, often subgrouped with standard doses in the analysis. This practice can potentially skew the results and limit the comparability of findings across studies. This lack of standardization in dose ranges represents a significant gap that future research needs to address. By standardizing dose ranges, future studies can ensure more reliable and comparable findings, thereby advancing our understanding of the anticancer efficacy of natural coumarins such as AUR and UMB. Moreover, it’s important to note that in vitro results may not entirely mirror clinical outcomes. A crucial aspect to consider is the safety profile of AUR and UMB. According to published studies, both agents exhibit minimal systemic toxicity and are well-tolerated in animal models at doses demonstrating chemopreventive or chemotherapeutic efficacy^31,39,41,52,53,56–59^. Furthermore, data from other in vivo studies and a human clinical trial highlight the favorable biosafety and protective effects of AUR^55,60–63^. However, there is a lack of direct comparative safety assessments, particularly concerning long-term impacts. Future research should investigate the differential effects of therapeutic versus supra-therapeutic doses on organ function and biochemical/hematological parameters over prolonged periods. Determining safety margins and the nature of potential adverse events would offer crucial additional insights. Finally, publication biases cannot be fully excluded despite fail-safe N, indicating a possibility of type I errors in determining significance.

This meta-analysis, through the compilation and synthesis of existing evidence, identifies AUR and UMB as potential anticancer agents of interest. It provides much-needed insight into the key characteristics that underpin their activity. To further these findings, it is advisable to undertake supplementary animal studies and concentrate on the pharmacokinetic properties of these compounds. The knowledge acquired from this research can steer future investigations to address their study more effectively. Given the wealth of data derived from in vivo models that closely resemble human cancers, the stage is set for the design of clinical trials. While individual molecules may necessitate further scrutiny, the results imply that plant extracts rich in AUR and UMB could be investigated as beneficial dietary or medicinal plant supplements for cancer therapy. This marks an exciting step forward in the field of natural anticancer therapeutics.

## Data Availability

The data that support the findings of this study are available on request from the corresponding author.
